# Saunders’s Medical Hand-Atlases

**Published:** 1901-09

**Authors:** 


### BOOK REVIEWS.

SAUNDERS'S MEDICAL HAND-ATLASES. Atlas and Epitomé of Ophthalmoscopy and Ophthalmoscopic Diagnosis. By PROFESSOR DR. O. HAAB, Director of the Eye Clinic in Zurich. From the third revised and enlarged German edition. Edited by George E. de Schweinitz, Professor of Ophthalmology, Jefferson Medical College, Philadelphia. With 152 colored lithographic illustrations and 85 pages of text. Philadelphia and London: W. B. Saunders & Co. 1901. [Price, $3.00 net.]

This is a tianslation from the third German edition and it is one of the most valuable of the series. Its value is increased by the fact that it was edited by Dr. G. E. de Schweinitz, of Philadelphia, which in itself is a guaranty that it is intelligible and that the plate work is beyond reproach. These ophthalmoscopic drawings are exquisitely executed, and in many cases microscopic lesions are shown. To those students who are beginning or are about to begin ophthalmic work, this book is simply invaluable. It is as necessary to him as is his knowledge of the anatomy of the eye, for it is the best work of its kind published and nowhere is there a more accomplished teacher and ophthalmologist than Dr. Haab.

Dr. de Schweinitz has edited the translation with a liberal hand and a confidence in his own knowledge which has been the means of adding to the value of the book. He has made comments freely throughout the text and to the illustrations he has added some from his own wide and varied experience. The book is not alone of use to students. Some of the teachers of ophthalmology might with great benefit study its style. It may not materially increase their personal knowledge of the subject, but it will make them better teachers. N.W.W.

